# PARP12 is required for mitochondrial function maintenance in thermogenic adipocytes

**DOI:** 10.1080/21623945.2022.2091206

**Published:** 2022-08-02

**Authors:** Fan Hu, Chang Li, Yafen Ye, Xuhong Lu, Miriayi Alimujiang, Ningning Bai, Jingjing Sun, Xiaojing Ma, Xiaohua Li, Ying Yang

**Affiliations:** a Shanghai Clinical Center for Diabetes, Shanghai Key Clinical Center for Metabolic Disease, Shanghai Diabetes Institute, Shanghai Key Laboratory of Diabetes Mellitus, Shanghai Jiao Tong University Affiliated Sixth People’s Hospital, China; b Department of Endocrinology, Seventh People’s Hospital Affiliated to Shanghai University of TCM, Shanghai, China

**Keywords:** Adipocytes, poly(ADP-ribose) polymerases, mitochondria, thermogenesis

## Abstract

PARP12 is a member of poly-ADP-ribosyl polymerase (PARPs), which has been characterized for its antiviral function. Yet its physiological implication in adipocytes remains unknown. Here, we report a central function of PARP12 in thermogenic adipocytes. We show that PARP12 is highly expressed in brown adipose tissue and is mainly localized to the mitochondria. Knockdown of PARP12 in vitro reduced UCP1 expression. In parallel, the deficiency of PARP12 reduced mitochondrial respiration in adipocytes, while overexpression of PARP12 reversed these effects.

## Introduction

Obesity has become a serious public health problem and poses a challenge to human health [1]. Adipose tissue plays a major role in the regulation of whole-body energy homoeostasis. White adipose tissue (WAT) is the major energy storage site. While brown adipose tissue (BAT) is an energy dissipation depot, which uncouples respiration from ATP synthesis and generates heat through a process called adaptive thermogenesis, owing to a high density of mitochondria and uncoupling protein 1(UCP1) [2,3]. In addition, in certain WAT depots exist brown-like adipocytes, also known as beige adipocytes, which emerge in response to cold acclimation, exercise training, or pharmacological activation of β-adrenergic receptors [4,5]. Activating brown or beige adipocytes may provide an effective means to prevent obesity and metabolic diseases. Mitochondria functions are vital for thermogenesis. Many studies showed that mitochondrial dynamics or mitochondrial respiration regulate thermogenesis in adipocytes [6,7]. Identifying novel regulators of mitochondrial function could lead to new strategies to promote thermogenesis.

The poly-ADP-ribosyl polymerase (PARPs) represent a family of enzymes, which use NAD+ as a substrate to modify target proteins by attaching ADP-ribose [8], and this process is defined as PARylation. Several mitochondrial proteins have been identified as the target of PARylation [9–13]. However, PARPs enzymatic activity mainly accounts for nuclear modifications, there is no direct evidence that PARPs mediated the PARylation of mitochondrial proteins. In addition, many studies have shown that PARPs can exert its function independent of its enzymatic activity [14,15]. The most known function regulated by PARPs is related to cellular stress response, such as DNA repair, and the PARP enzyme inhibitors are in clinical trials as a treatment for tumours. There are 17 PARPs members in humans and 16 PARPs in mice [8]. PARP1 and PARP2 are the most characterized PARP family member, contributing to 90% of cellular PARP activity [16]. Several studies indicated that the involvement of PARP1 and PARP2 in metabolic regulation and disease. Recent investigations have enlarged our view, implying that other members of the PARP family function in metabolic regulation as well [17–19]. PARP12 has been identified as a putative antiviral gene belonging to an interferon-stimulated gene [20,21]. PARP12 contains typical CCCH zinc finger domains, suggesting that it may play an important role in RNA processing [22]. Accordingly, it has been found that PARP12 accumulated in cytoplasmic stress granules, known sites of mRNA translational arrest, and is involved in regulating protein translation and inflammation [23]. However, the function of PARP12 in adipocytes remains largely unknown.

In this study, we aim to explore the role of PARP12 in thermogenic fat cells. We found that PARP12 is enriched in BAT and mainly localized to the mitochondria. PARP12 deficiency suppressed UCP1 expression. The deficiency also modestly reduced mitochondrial respiration in adipocytes. Overall, our results may suggest that PARP12 plays an important role in maintaining mitochondrial respiration and UCP1 expression.

## Materials and methods

### Animals

Mice were housed in 12 h light/dark cycle and given free access to food and water. For cold stimulation, mice were subjected to 4°C for 24 h or 7 days. CL-316243 (Sigma, C5976) (1 mg/kg) were implanted subcutaneously with mini-pumps for 7 days. The swimming exercise was implemented as previously described [24]. All animal studies were approved by the Animal Care Committee of Shanghai Jiaotong University School of Medicine.

### Human adipose tissue samples

Human subcutaneous and deep neck adipose samples were obtained from patients scheduled for thyroidectomy surgery, which have previously been described [24].

### Isolation and culture of primary adipocytes

To culture brown or beige adipocytes, stromal vascular fraction (SVF) was isolated from iWAT and BAT of mice as previously described [25]. After reaching confluence, cells were differentiated in growth medium supplemented with 0.5 mM IBMX (Sigma, I7018), 1 μM rosiglitazone (Sigma, R2408), 1 nM T3 (Sigma, T2877), 1 μM dexamethasone (Sigma, D4902), and 5 μg/ml insulin (Lily, HI0240) for 2 days (day 0 to day 2), and then maintained in medium with rosiglitazone, T3 and insulin for 4 days (day 2 to day 6). In some experiments, cells were treated with 0.5 mM dibutyryl-cAMP (Sigma, D0627) or 20 μM H-89 (Sigma, B1427) and 10 μM SB202190 (Sigma, S7067). Isoproterenol was added to medium for the last 6 h during culture.

### Oil Red O staining

Cells were rinsed twice with PBS and fixed in 4% paraformaldehyde for 15 min. Then, cells were washed twice with PBS and stained with Oil Red O working solution (Sigma-Aldrich, O1391) for 10–20 min at room temperature. Next, stained cells were rinsed with PBS three times. The lipid droplets were evaluated by the fluorescence microscope (Nikon Corp, Japan) and representative figures were shown.

### siRNA-mediated knockdown

On day 0 or day 5, cells were reverse-transfected with siRNA using RNAiMAX (Invitrogen). For 24-well plates, 1.5ul siRNA (20uM) was dissolved in 25ul Opti-MEM reduced serum medium (Invitrogen), 2.5ul RNAiMAX was diluted in 25ul Opti-MEM. We then add the diluted siRNA to diluted RNAiMAX solution, incubate at RT for 5 min. Finally, the siRNA mix was added to the cells. Cells were collected 48–72 h after transfection. The siRNA sequences target for PARP12 and negative control (NC) were as follows: siPARP12: 5’-GCAGGCUACUCUCUACUUATT-3’, siNC: 5’-UUCUCCGAACGUGUCACGUTT-3’, which were designed and synthesized by Gene Pharma (Shanghai, China).

### Lentivirus transduction

A lentivirus containing the PARP12 expression vector was packaged by the Shanghai Genechem Corporation. On differentiation day 3, cells were infected with PARP12 overexpressing or negative control (NC) lentivirus, with a multiplicity of infection of 50. Cells were harvested on day 8 for functional evaluation.

### Extracellular respiration

On day 6–8 mature adipocytes were loaded to an XF96 Extracellular Flux Analyser (Agilent). Mitochondrial respiration rate was quantified using the Mito-stress test protocol. In brief, on the day of the experiments, cells were washed three times and maintained in XF assay medium. Oligomycin (2 μM) was injected to inhibit mitochondrial ATP synthesis. FCCP was added to a final concentration of 2uM to quantify the maximum respiratory capacity of adipocytes. Antimycin A/rotenone was used to inhibit mitochondrial respiration and estimate the contribution of nonmitochondrial respiration to the measured OCR.

### Gene expression analysis (RT–qPCR)

Total RNA was extracted from tissues or cells using Trizol reagent (Invitrogen, 15,596,018). A total of 1ug RNA was reverse-transcribed using the Primer Script RT reagent Kit (Takara, RR047B). RT-qPCR was performed in a 384-well format using SYBR Premix Ex Taq (Takara, RR820A) with a CFX384 Real-time PCR system (Bio-rad). The relative mRNA expression was calculated using the ΔΔCt method and normalized to that of 36B4 mRNA as the reference gene. Primer sequences used for RT–PCR are listed in Table 1.

**Table 1.** RT-qPCR primer sequences.

Gene	Primer sequence (5’-3’)
*Parp12*	F: CTGGAGCAGTTGGAAAGGTTGGG R: GCGGGAGAAGGAGACACTTTGC
*Fabp4*	F: AAGGTGAAGAGCATCATAACCCT R: TCACGCCTTTCATAACACATTCC
*Pparg*	F: TCGCTGATGCACTGCCTATG R: GAGAGGTCCACAGAGCTGATT
*Ppargc1a*	F: TATGGAGTGACATAGAGTGTGCT R: CCACTTCAATCCACCCAGAAAG
*Adrb3*	F: TCTCTGGCTTTGTGGTCGGA R: GTTGGTTATGGTCTGTAGTCTCG
*Ucp1*	F: AGGCTTCCAGTACCATTAGGT R: CTGAGTGAGGCAAAGCTGATTT
*36B4*	F: AAGCGCGTCCTGGCATTGTCT R: CCGCAGGGGCAGCAGTGGT
*Dio2*	F: CAGTGTGGTGCACGTCTCCAATC R: TGAACCAAAGTTGACCACCAG
*H-Parp12*	F: GTACAGAACCTGGCCCTCTG R: GACCCGCCAGTCAAAGTTCT
*H-Ucp1*	F: GTGTGCCCAACTGTGCAATG R: CCAGGATCCAAGTCGCAAGA
*H-Rplp0*	F: AGCCCAGAACACTGGTCTC R: ACTCAGGATTTCAATGGTGCC
*mt-Rnr1*	F: AGGAGCCTGTTCTATAATCGATAAA
	R: GATGGCGGTATATAGGCTGAA
*Rbm15*	F: GGACACTTTTCTTGGGCAAC
	R: AGTTTGGCCCTGTGAGACAT

### RNA-seq

Total RNA from beige adipocytes were prepared using Total RNA Kit (Tiangen#DP419). Libraries were generated using VAHTSTM mRNA-seq V2 Library Prep Kit (Vazyme). cDNA libraries were pair-end sequenced on an Illumina HiSeq 6000. Using Hisat2 software, reads were aligned to the mouse genome GRCm38.100. EdgeR package was used for identifying differentially expressed genes (DEGs), with |Fold change|>1.5 and p-value <0.05 considered significant.

### Protein extraction and western blot analysis

Protein samples were isolated from adipose tissues and cells with RIPA buffer supplemented with protease inhibitor cocktail (Roche, 04693132001) and Phosphatase Inhibitor (Roche,4,906,845,001). The homogenates were centrifuged at 12,000 g for 20 min at 4°C, and the supernatants were used for subsequent analyses. Protein concentration was determined using the BCA protein assay kit. Protein samples were separated by SDS–PAGE on 10–12% polyacrylamide gels and transferred onto a nitrocellulose membrane. Membranes were incubated with indicated primary antibodies and then with secondary antibodies coupled to HRP. Primary antibodies were used, including PARP12 (Abcam, ab241967, dilution: 1:10,000, total protein 10ug), UCP1 (Abcam, ab10983, dilution: 1:2000 for cells, 1:5000 for tissues, total protein 10ug), total OXPHOS (Abcam, ab110413, dilution: 1:300, total protein 6ug), Tubulin (Sigma, T6199, dilution: 1:1000) For secondary-antibody incubation, anti-rabbit or anti-mouse HRP (CST) was diluted at 1:2000. Chemiluminescent signals were detected by the Image Quant LAS4000 Imaging systems (GE Healthcare).

### Transmission electron microscopy (TEM)

For TEM analysis of adipocytes, cell precipitation was collected and fixed in glutaraldehyde, followed by pre-embedding in 1% agarose, postfixed with 1% OsO4, dehydrated at room temperature, resin penetration and embedding, polymerization, section, and staining. Images were acquired using a HITACHI Transmission Electron Microscope.

### Mitochondrial DNA Content

Genomic DNA was extracted from cultured adipocytes using the Quick-DNA™ Miniprep Plus Kit (ZYMO, D4068). Genomic DNA was subjected to qPCR with primers for mt-RNR1 and RBM15 to measure mtDNA and nuclear DNA content, respectively, and calculate the Mt/N DNA ratio.

### MitoTracker staining

MitoTracker Green was added into the culture media at final concentrations of 100 nM, incubated at 37°C for 30 min, washed twice with PBS, and then visualized by fluorescence microscopy.

### Mitochondrial isolation

Brown adipose tissue or mature adipocytes were collected, and mitochondrial fractions were extracted using a mitochondrial isolation kit according to the manufacturer’s instructions (Beyotime, C3606, China). Protein was quantified using the bicinchoninic acid method.

### Statistical analyses

Data were expressed as mean ± sem. Statistical analysis was performed using a two-tailed Student’s t-test. The statistical significance was defined as *P* < 0.05, and expressed as *P < 0.05, **P < 0.01, ***P < 0.001.

## Results

### PARP12 is enriched in thermogenic fat cells

**Figure 1.**
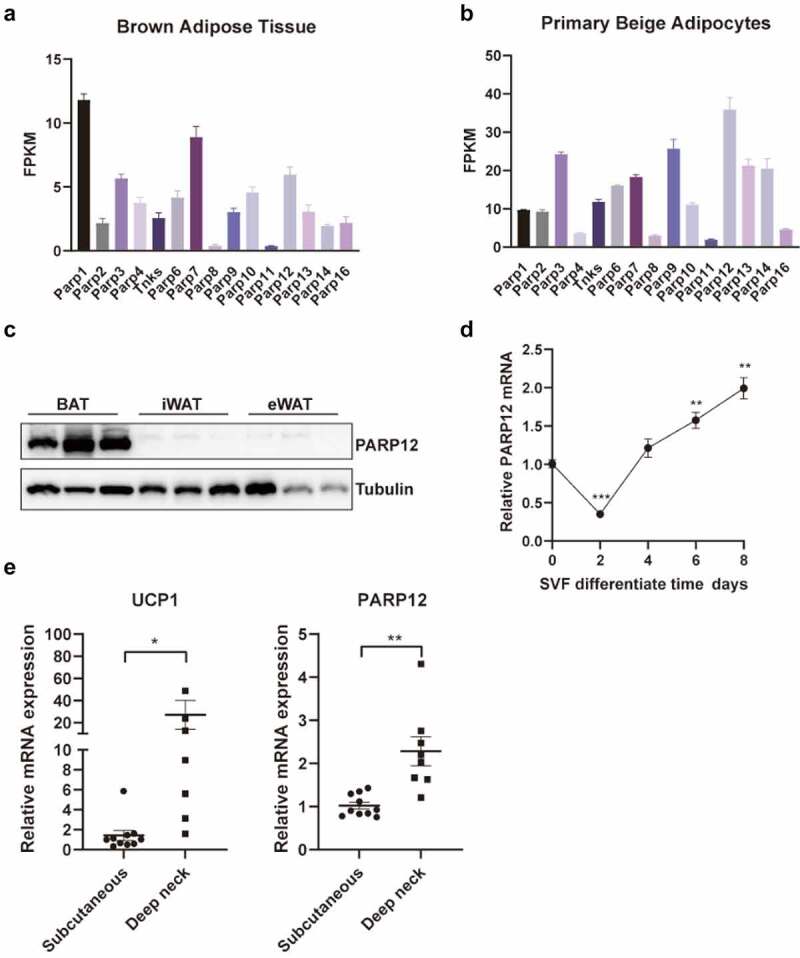
**Expression of PARP12 in adipose tissue and adipocytes**. (a-b) The reads of PARP family members in BAT and primary beige adipocytes from RNA-seq. FPKM: fragments per kilobase of exon per million reads. (c) PARP12 protein level in brown and white adipose tissue of C57/BL6 male mice. (d) PARP12 level in differentiating svf derived primary beige adipocytes. (e) RT-qPCR analysis of PARP12 and UCP1 mRNA level in human deep neck fat and subcutaneous fat (n = 7–10), UCP1 is shown as a positive control. The data shown are mean ± SEM. *P < 0.05, **P < 0.01 and ***P < 0.001.

First, we queried our previous RNA sequencing (RNA-seq) data to identify the involvement of PARP12 in adipocytes. In BAT, PARP1 expression was the highest among all the PARP family members (Figure 1(a)), while in beige adipocytes, PARP12 was higher than that of the other members (Figure 1(b)). We next examined PARP12 expression in adipose tissues. PARP12 expression was significantly higher in BAT, compared with inguinal WAT (iWAT) and epididymal WAT (eWAT)(Figure 1(c)). Moreover, we isolated SVFs from the iWAT of mice and differentiated them to beige adipocytes. PARP12 expression declined after 2 days of induction and increased thereafter (Figure 1(d)). These results suggested the potential role of PARP12 in thermogenic adipocytes. Furthermore, We sought to determine whether this could be relevant to humans. As adult human deep neck adipose tissue has molecular signatures of classical BAT, we compared PARP12 levels in human subcutaneous and deep neck adipose depots. We observed that the mRNA expression of PARP12 was higher in deep neck fat than in the subcutaneous fat, with a similar change of the thermogenic marker UCP1 (Figure 1(e)).

### Adipose PARP12 expression is regulated by thermogenic stimuli

**Figure 2.**
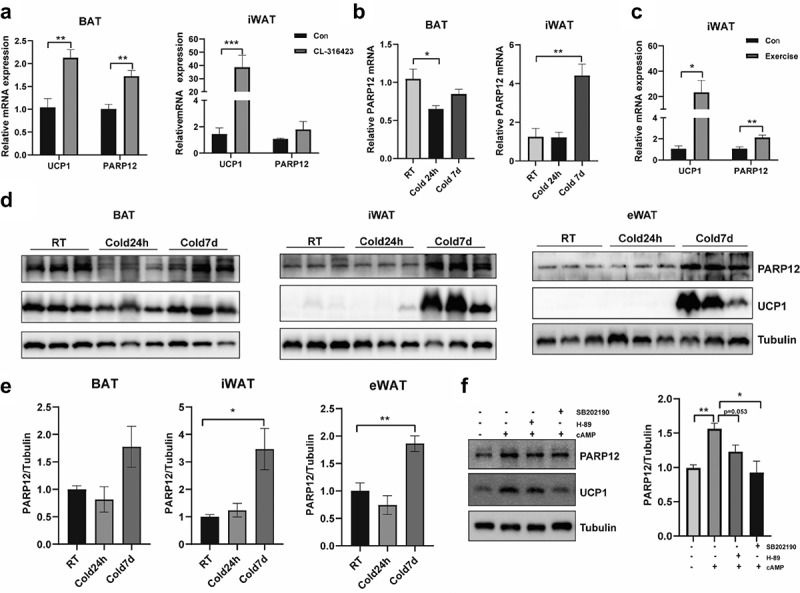
**Adipose PARP12 expression is induced by β-adrenergic signalling**. (a) PARP12 mRNA level in BAT and iWAT after the treatment of CL316423 (n = 4–6). (b) PARP12 mRNA level in BAT and iWAT after cold exposure for 24 hours and 7 days (n = 6–10). (c) PARP12 mRNA level in iWAT from exercise-trained mice (n = 8). (d) The PARP12 protein level in BAT, iWAT, and eWAT after cold challenge for 24 hours and 7 days (n = 3). (e) Quantification of PARP12 protein level in (d). (f) Effects of PKA inhibitor (H89) and p38-MAPK inhibitor (SB202190) on the change of PARP12 and UCP1 protein level in beige adipocytes when cAMP stimulated for 12 h, and the quantification of PARP12 protein level. Data were presented as mean ± SEM. *P < 0.05, **P < 0.01 and ***P < 0.001.

We next assessed whether PARP12 might be induced during thermogenesis. Activation of β3-adrenergic receptor by CL316243 agonist-induced PARP12 expression in BAT and a moderate increase in WAT (Figure 2(a)). Cold exposure also activates thermogenesis. To explore the role of PARP12 in cold-induced thermogenesis, mice were maintained at 4°C for 24 h or 7d. The expression of PARP12 was slightly decreased in BAT or remained unchanged in WAT when mice were subjected to acute cold exposure, while the PARP12 transcript and protein levels were significantly induced in WAT after chronic cold exposure(Figure 2(b,d-e)). Similarly, higher PARP12 mRNA expression was observed in iWAT from exercise-trained mice, suggesting PARP12 may be also involved in the process of white adipose browning(Figure 2(c)). These results indicated that the expression of PARP12 is induced by thermogenic activation. Next, we treated adipocytes with cAMP, which enhanced the activity of the PKA and p38 mitogen-activated protein kinase (p38 MAPK) pathway. Results showed that PARP12 protein level increased after the treatment of cAMP, while blocked by the PKA antagonist H89 and the p38-MAPK antagonist SB202190, similar to the UCP1 level(Figure 2(f)), suggesting that PARP12 might be regulated by the cAMP/PKA/p38 MAPK pathway.

### The effect of PARP12 on UCP1 expression

First, we explored the role of PARP12 in adipocyte differentiation; SVFs derived from iWAT were utilized to induce adipocytes in vitro. Preadipocytes were treated with either negative control (NC) siRNA or siRNA targeting PARP12 to knockdown. Quantitative RT-PCR confirmed the PARP12 expression decreased significantly (Figure S1A). PARP12 reduction in preadipocytes affected the mRNA expression levels of common adipogenic-related markers, including Fabp4, CEBPα, and PPARγ (Figure S1A). The Oil Red O staining results showed that PARP12 knockdown caused a detectable difference in lipid accumulation on day 6 during the differentiation programme(Figure S1B). Together, these results suggested that the loss of PARP12 expression prevents adipocyte differentiation.

**Figure 3.**
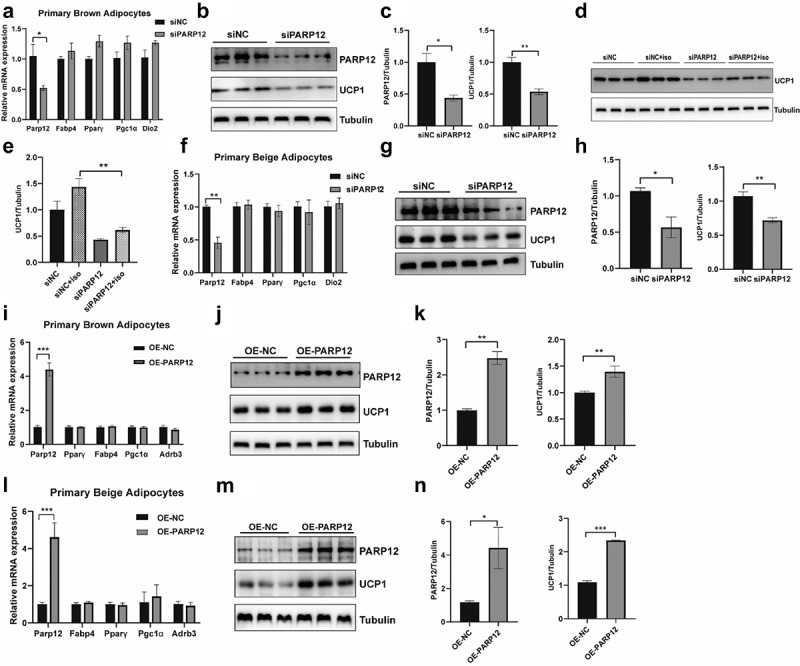
**The effect of PARP12 on UCP1 expression**. The mRNA level of indicated genes in brown adipocytes (a) and beige adipocytes(f) after PARP12 knockdown (n = 4). PARP12 and UCP1 protein levels in brown adipocytes (b) and beige adipocytes (g) after PARP12 knockdown (n = 3). (c) and (h) Quantification of PARP12 and UCP1 protein level. (d-e) Basal or iso-stimulated UCP1 expression in brown adipocytes (n = 3). The mRNA level of indicated genes in brown adipocytes (i) and beige adipocytes (l) after PARP12 overexpressed (n = 5). PARP12 and UCP1 protein levels in brown adipocytes (j) and beige adipocytes (m) after PARP12 overexpressed (n = 3). (k) and (n) Quantification of PARP12 and UCP1 protein levels in **(j)** and (m). Data were presented as mean ± SEM *P < 0.05, **P < 0.01 and ***P < 0.001.

We also asked whether PARP12 might affect adipocyte function outside of the context of differentiation. We, therefore, performed loss of function experiments on mature brown adipocytes (day 5). Reduction of PARP12 in mature adipocytes did not affect the mRNA expression levels of differentiation-related genes (Figure 3(a)) but repressed the protein level of UCP1 compared to the siNC group(Figure 3(b-c)), and a more pronounced reduction was observed when cells were stimulated with isoproterenol (ISO) (Figure 3(d-e)). To determine whether the decrease in UCP1 after PARP12 loss was specific to brown adipocytes, we differentiated beige adipocytes from iWAT of mice and ablated the expression of PARP12. We observed a significant decrease in the level of UCP1 (Figure 3(g-h)), with no alteration of adipogenic marker expression (Figure 3(f)). To address the question of whether an increase in PARP12 expression would cause the opposite phenotype, we infected mature adipocytes with a lentivirus that overexpresses PARP12. The infection led to a fourfold overexpression of PARP12 and an increase in UCP1 expression(Figure 3(i-k)). Overexpression of PARP12 consistently did not alter the mRNA levels of any of the adipogenic-related genes(Figure 3(i)). Similarly, we also observed the increased UCP1 expression in beige adipocytes with no alteration of PPARγ and Fabp4 expression (Figure 3(l-n)).

### PARP12 is important for mitochondrial respiration in thermogenic adipocytes

**Figure 4.**
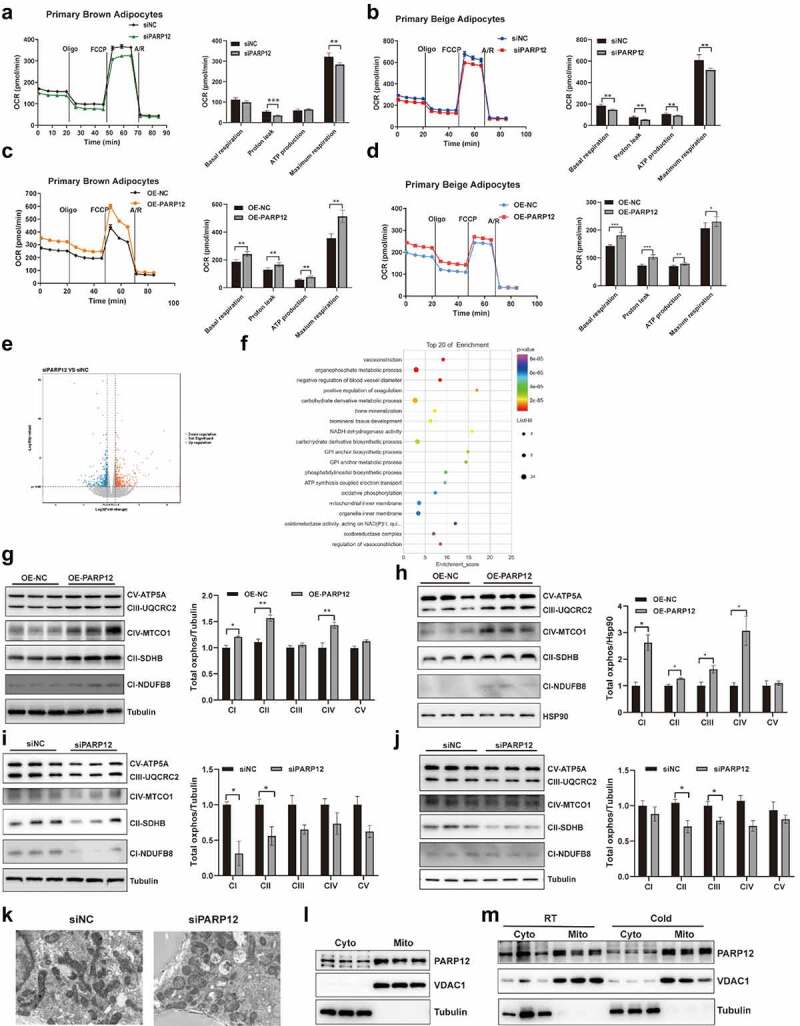
**The effect of PARP12 on mitochondrial function**. Oxygen consumption rate was measured in PARP12 knockdown brown adipocytes (a) and beige adipocytes (b) (n = 6–8). Oxygen consumption rate was measured in PARP12 overexpressing brown adipocytes (c) and beige adipocytes (d). (e) Volcano plots of RNA-seq (n = 3). (f) Go terms enriched for the differentially expressed genes. (g-h) Western blot analysis showing OXPHOS complexes expression in brown and beige adipocytes overexpressed PARP12. (i-j) OXPHOS complexes expression in brown and beige adipocytes transfected with siPARP12 and siNC (n = 3). (k) Representative TEM images of mitochondrial structure in beige adipocytes transfected with siPARP12 and siNC. (l) Western blot of the indicated fraction (n = 3). (m) BAT was collected from mice at 4°C and immunoblotting in indicated fraction (n = 3).

To determine the effect of PARP12 on mitochondrial function, we evaluated oxygen consumption rate (OCR). The results showed that PARP12 knockdown cells had lower respiration, as the cellular metabolic parameters, including proton leak and maximal respiration were decreased (Figure 4(a-b)). We also observed that the oxygen consumption increased in PARP12 overexpressing cells (Figure 4(c-d)).

To reveal a possible regulatory mechanism, we employed RNA-seq in PARP12 knockdown cells. As shown in Figure 4(e), a total of 272 differentially expressed genes (DEGs) were detected, such as mt-ND2, mt-ND4, mt-ATP6, and Uqcc3. Gene ontology (GO) analysis showed that these genes were enriched in NADH dehydrogenase activity, ATP synthesis coupled electron transport, oxidative phosphorylation, and mitochondrial inner membrane (Figure 4(f)). We therefore next evaluated the mitochondrial complex in PARP12 knockdown and overexpressed cells by western blot. As result, we found PARP12 overexpression enhanced the level of complexI, II and IV both in brown and beige adipocyes (Figure 4(g-h)), while PARP12 reduction lead to a lower protein abundance for complex I and II in brown adipocytes, and decreased complex II and III in beige adipocyes (Figure 4(i-j)) Transmission electron microscopy (TEM) imaging showed that mitochondria were large and swollen, with no distinct change of cristae structure in PARP12 knockdown cells (Figure 4(k)). In addition, we observed no change of mitochondrial number as reflected by mitochondria DNA/genomic DNA ratio and MitoTracker Green(Figure 2(a-b)). Moreover, we separated the cytosolic fraction without mitochondrial contamination and found that PARP12 was more abundant in the mitochondrial fraction (Figure 4(l)). When mice were exposed at 4°C, PARP12 was detected at a higher level in mitochondrial fraction and a lower level in the cytosolic fraction (Figure 4(m)). These results implicated that PARP12 is mainly localized in mitochondria and is required in regulating the components of the oxidative phosphorylation chain.

## Discussion

Although originally described as DNA damage repair agents, recent data highlight novel roles for PARP enzymes in metabolic regulation. In this study, we found that PARP12 is highly expressed in BAT and is induced by the thermogenic stimulus. PARP12 deficiency reduced UCP1 expression. Also, PARP12 deficiency suppressed mitochondrial respiration. Whereas overexpressing PARP12 reverses these effects. Our results suggest that PARP12 is important for maintaining mitochondrial function in thermogenic adipocytes.

Previous studies reported that PARP1 supports adipocyte differentiation [26,27], in contrast, recent studies show that inhibition or depletion of PARP1 promotes the adipogenic differentiation programme [28–30]. In our study, knockdown of PARP12 in preadipocytes repressed adipogenic related gene expression, suggesting that PARP12 repressed differentiation to some extent. There is no explanation for the discrepancies, and the Bai group [16] even observed different clones of the 3T3-L1 cells have different behaviour in differentiation and response to PARP inhibitors. Considering that PARP12 might affect adipogenesis, we knockdown or overexpressed PARP12 in post-differentiation adipocytes, which could avoid affecting differentiation by PARP12 as much as possible. The results showed that loss of PARP12 in adipocytes did not alter the adipogenic marker (Fabp4 and Pparγ) expression, but indeed altered the UCP1 levels.

Most of the cellular PARP activity is localized to the nucleus, the cytoplasm is also known to harbour PARP activity. It has been reported that PARP activity may exist in mitochondria, as studies show that the presence of PARylated proteins in mitochondria or PAR-degrading activity appears to be present in mitochondria [31–34], however well-defined PARP activity or its presence is still debatable. PARP12 has a relatively homogenous, cytoplasmic distribution in fibroblast, upon a specific signal, it can relocate to stress granules or p62-containing structures [23]. Considering that different cellular distributions of PARPs may indicate their distinctive functions, we examined the localization of PARP12 and found that PARP12 is mainly localized in mitochondria in adipocytes. Moreover, upon cold exposure, PARP12 is detected at a higher level in the mitochondrial fraction. Thus, we speculate that PARP12 plays a vital role in mitochondrial function.

It has been reported that activation of PARPs impaired mitochondrial function. Genetic PARP1 inhibition was shown to induce mitochondrial biogenesis [35]. Olaparib, which is a selective PARP1/2 inhibitor, induced mitochondrial biogenesis in white adipocytes and enhanced UCP1 expression [36]. In the latter, mitochondrial biogenesis upon Olaparib treatment is governed by PARP1 and PARP2. Our study indicated that PARP12 may have a different role from PARP1, as the evidence of its localization and function on mature adipocytes. Mitochondrial functions, including fatty acid oxidation, tricarboxylic acid cycle, electron transport chain, are all required for UCP1 mediated adaptive thermogenesis in adipocytes. Although deletion of PARP12 resulted in repressed UCP1 level and impaired mitochondrial function, it has not been determined whether PARP12 directly affects UCP1 or if this is just a consequence of the impaired mitochondrial function.

The underlying mechanisms of PARP12 modulate mitochondrial function are still unknown. PARPs can modulate mitochondrial function by interacting with Sirtuins, HIFs, or mitochondrial PARylation [37]. Thus, identifying the PARP12-interacting mitochondrial proteins would help us to understand the underlying mechanisms. Some pathways that are independent of PARylation may also participate in maintaining mitochondria homomasis, in light of not all PARPs family members exert its role by PARylation [38,39]. Besides, given that some RNA binding proteins (RBPs) have been identified as essential for adipogenesis and thermoregulation [40–42], as an RBP, PARP12 may also exert its function by affecting RNA processes and post transcription. Thus, further investigations are needed to explore the potential mechanisms.

Taken together, we reveal that PARP12 plays an important role in thermogenic fat cells and provides new knowledge for PARP12 in mitochondrial function maintenance. Further studies are needed to fully characterize the role of PARP12 in system metabolism in vivo and elucidate the possible mechanisms involved.

## Supplementary Material

Supplemental Material

## Data Availability

All data that support the findings of this study are available within the article, its Supplementary Information, or from the corresponding author upon reasonable request.
