# Supplementary material for: PARP12 is required for mitochondrial function maintenance in thermogenic adipocytes
Source: Adipocyte. 2022 Aug 2;11(1):379–88. doi: 10.1080/21623945.2022.2091206 (PMC9351573; doi:10.1080/21623945.2022.2091206)
Supplement: Supplemental Material [file KADI_A_2091206_SM1770.docx]

**
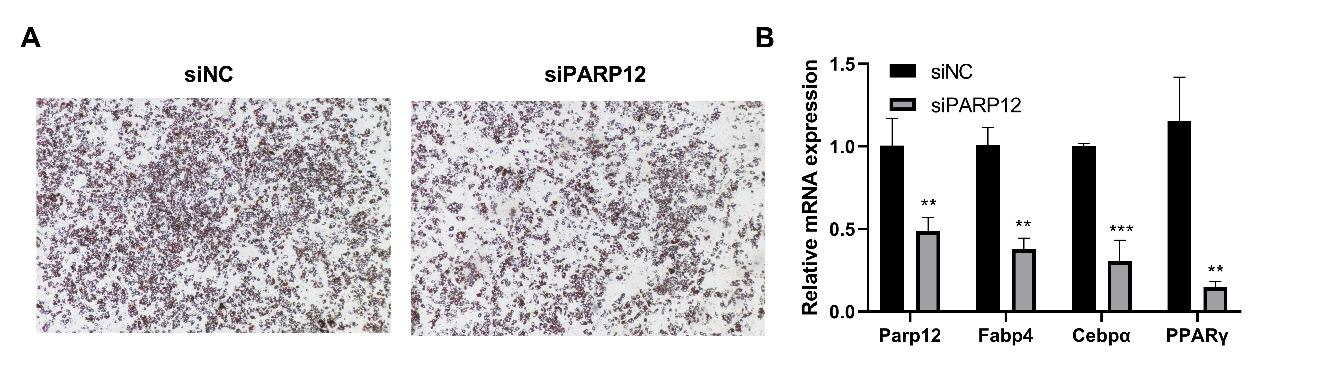
**

**Fig.S1 The effect of PARP12 on adipocyte differentiation. (A)** Oil Red O staining shows lipid accumulation in siNC and siPARP12 adipocytes. **(B)** Common differentiation-related genes expression was detected by quantitative RT-PCR. ***P* < 0.01 and ****P* <0.001.


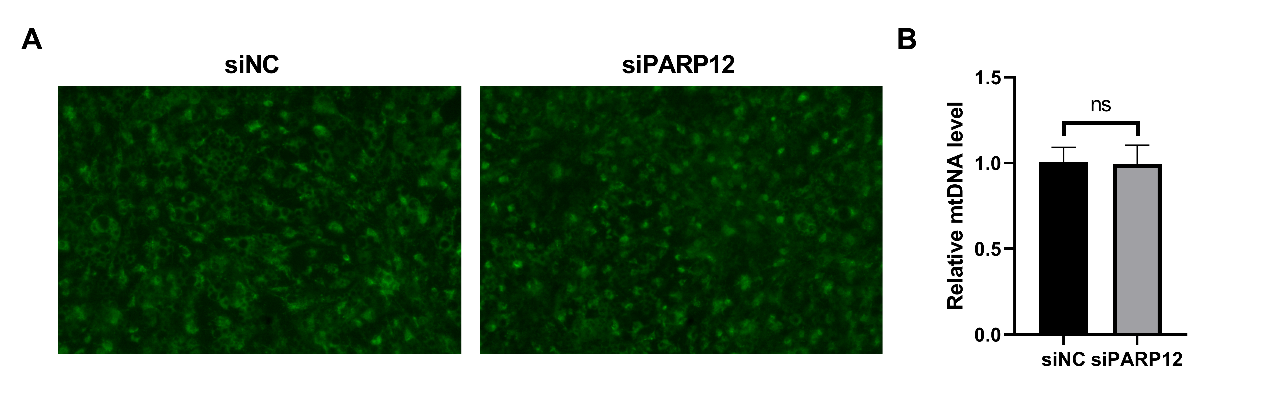


**Fig.S2 The effect of PARP12 on the mitochondrial number. (A)** Representative fluorescent images of Mito tracker staining in beige adipocytes differentiated from iWAT SVF cells. **(B)** Mitochondrial DNA to nuclear DNA ratio in beige adipocytes(n=4).
